# Author Correction: Genomics and biochemical analyses reveal a metabolon key to β-L-ODAP biosynthesis in *Lathyrus sativus*

**DOI:** 10.1038/s41467-023-40984-6

**Published:** 2023-08-25

**Authors:** Anne Edwards, Isaac Njaci, Abhimanyu Sarkar, Zhouqian Jiang, Gemy George Kaithakottil, Christopher Moore, Jitender Cheema, Clare E. M. Stevenson, Martin Rejzek, Petr Novák, Marielle Vigouroux, Martin Vickers, Roland H. M. Wouters, Pirita Paajanen, Burkhard Steuernagel, Jonathan D. Moore, Janet Higgins, David Swarbreck, Stefan Martens, Colin Y. Kim, Jing-Ke Weng, Sagadevan Mundree, Benjamin Kilian, Shiv Kumar, Matt Loose, Levi Yant, Jiří Macas, Trevor L. Wang, Cathie Martin, Peter M. F. Emmrich

**Affiliations:** 1 John Innes Centre, Norwich Research Park, Colney Lane, Norwich, NR4 7UH UK; 2 Biosciences eastern and central Africa International Livestock Research Institute Hub, ILRI campus, Naivasha Road, P.O. 30709, Nairobi, 00100 Kenya; 3 Queensland University of Technology, 2 George St, Brisbane City, QLD 4000 Australia; 4 National Institute of Agricultural Botany, 93 Laurence Weaver Road, Cambridge, CB3 0LE UK; 5 School of Traditional Chinese Medicine, Capital Medical University, You An Men, Beijing, 100069 PR China; 6 Earlham Institute, Norwich Research Park, Colney Lane, Norwich, NR4 7UZ UK; 7 School of Life Sciences, University of Nottingham, University Park, Nottingham, NG7 2RD UK; 8 Institute of Plant Molecular Biology, Biology Centre CAS, Branisovska 31, Ceske Budejovice, CZ-37005 Czech Republic; 9 Research and Innovation Centre, Fondazione Edmund Mach, Via Edmund Mach 1, 38098 San Michele all’ Adige (TN), Italy; 10 Whitehead Institute for Biomedical Research, Cambridge, MA 02142 USA; 11 Department of Biological Engineering, Massachusetts Institute of Technology, Cambridge, MA 02139 USA; 12 Department of Biology, Massachusetts Institute of Technology, Cambridge, MA 02139 USA; 13 Global Crop Diversity Trust, Platz der Vereinten Nationen 7, 53113 Bonn, Germany; 14 International Center for Agricultural Research in the Dry Areas, Avenue Hafiane Cherkaoui, Rabat, Morocco; 15 Future Food Beacon of Excellence, University of Nottingham, NG7 2RD Nottingham, UK; 16 Norwich Institute for Sustainable Development, School of International Development, University of East Anglia, Norwich, NR4 7TJ UK

**Keywords:** Agricultural genetics, Genome, Secondary metabolism, Multienzyme complexes

Correction to: *Nature Communications* 10.1038/s41467-023-36503-2, published online 16 February 2023

The original version of this Article contained errors in the author affiliations. Affiliation 11 was incorrectly given as “Department of Biological Engineering, Whitehead Institute for Biomedical Research, Massachusetts Institute of Technology Cambridge, Cambridge, MA 02142-1479, USA” but should be “Department of Biological Engineering, Massachusetts Institute of Technology, Cambridge, MA 02139, USA”. Similarly, affiliation 12 was incorrectly given as “Department of Biology, Whitehead Institute for Biomedical Research, Massachusetts Institute of Technology Cambridge, Cambridge, MA 02142-1479, USA” but should be “Department of Biology, Massachusetts Institute of Technology, Cambridge, MA 02139, USA”. In addition, the original version of this Article omitted the acknowledgement text: “We thank Prof. Stephen Bornemann for his insight and advice on the enzymology and chemistry of β-L-ODAP formation”. Furthermore, symbols of enzyme turnover number were incorrectly given as “*Kcat*” in Table 2 of the original version of this Article. The correct symbol should be given as “*kcat*”. These have been corrected in both the PDF and HTML versions of the Article.

