# Supplementary material for: Genomics and biochemical analyses reveal a metabolon key to β-L-ODAP biosynthesis in Lathyrus sativus
Source: Nat Commun. 2023 Feb 16;14:876. doi: 10.1038/s41467-023-36503-2 (PMC9935904; doi:10.1038/s41467-023-36503-2)
Supplement: Supplementary file 1 — Supplementary information [file 41467_2023_36503_MOESM1_ESM.pdf]

Genomics and biochemical analyses reveal a metabolon key to β-L-ODAP biosynthesis in *Lathyrus sativus*

Edwards *et al.*

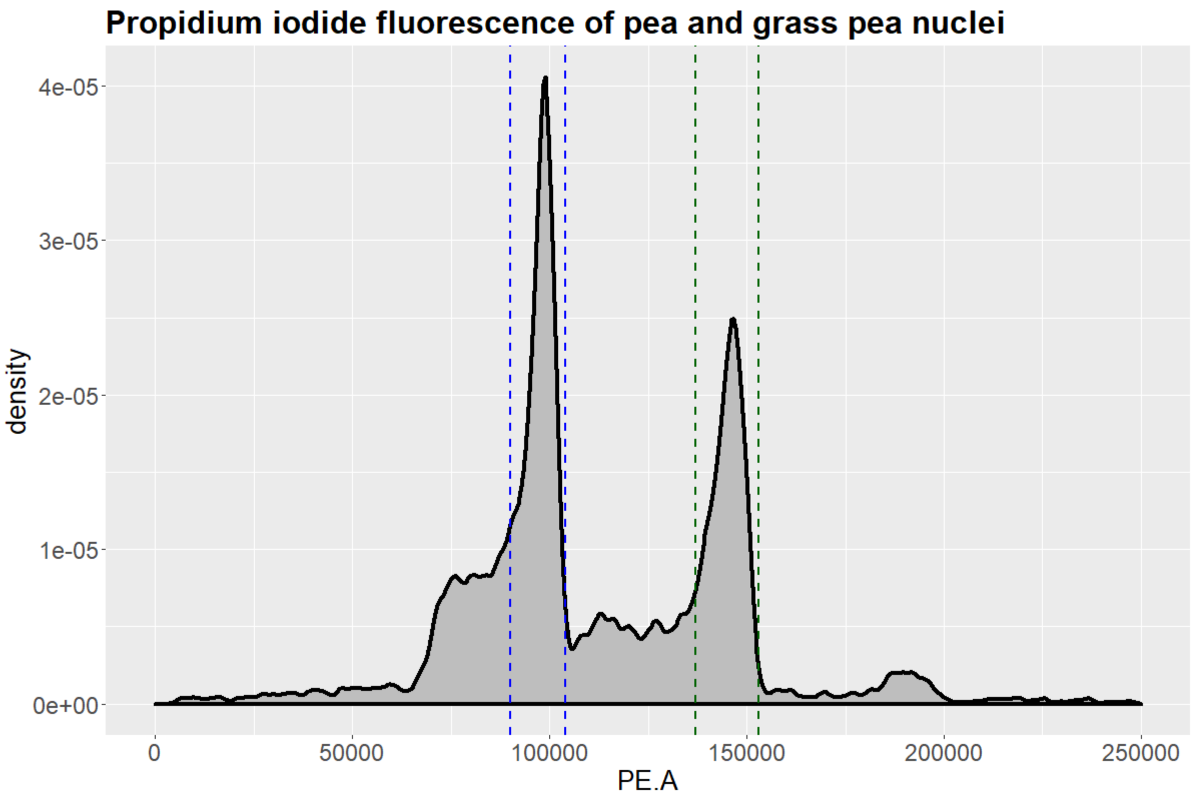

Supplementary Figure 1. Results of a representative flow cytometry run, after gating to exclude cell debris events. Propidium iodide fluorescence amplitude (in arbitrary units) is shown against event density. The interval assumed to be pea nuclei is defined by blue dashed lines, the interval assumed to be grass pea nuclei is defined by green dashed lines. The experiment was replicated three times. The gating strategy is shown in Supplementary Figure 11. Source data are provided as a Source Data file.

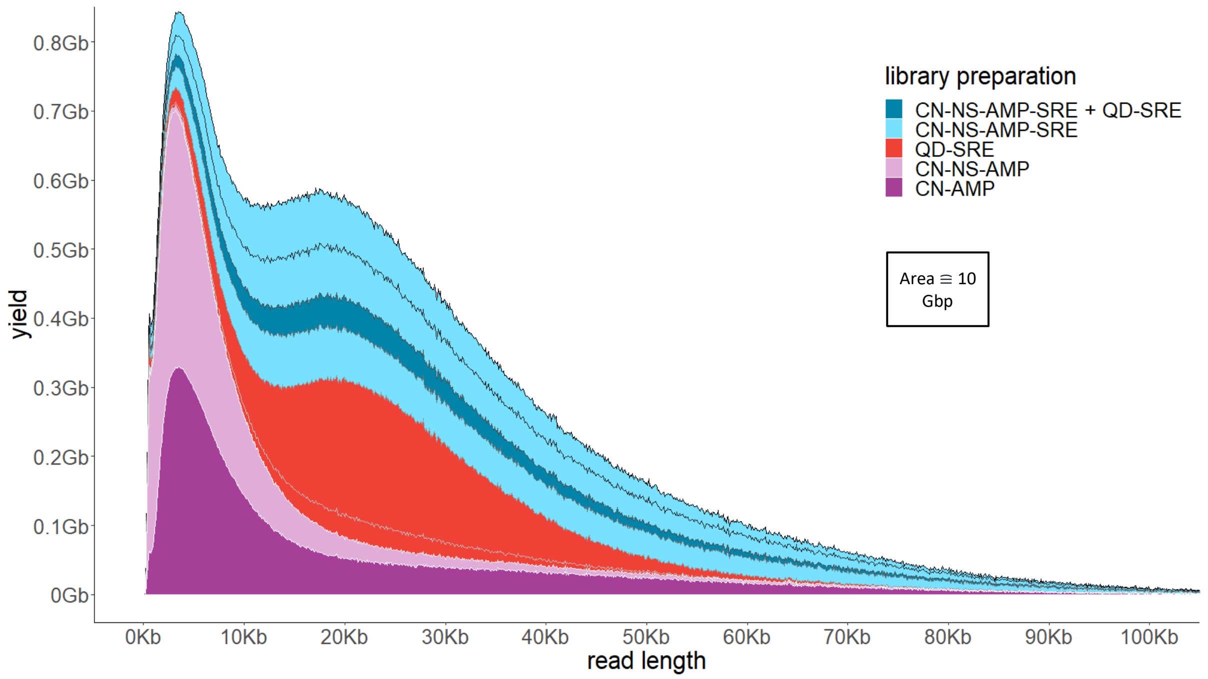

Supplementary Figure 2. Distribution of read lengths of Nanopore PromethION data, separated by library preparation procedure. Source data are provided as a Source Data file.

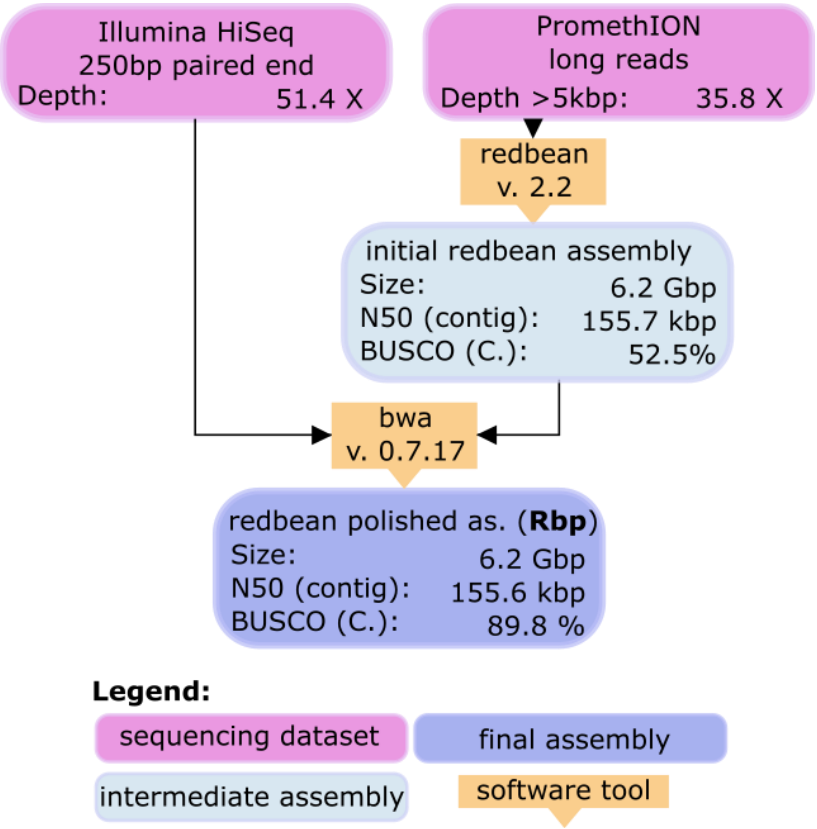

Supplementary Figure 3. Workflow of assembly of Rbp from ONT data and polishing with Illumina paired-end data.

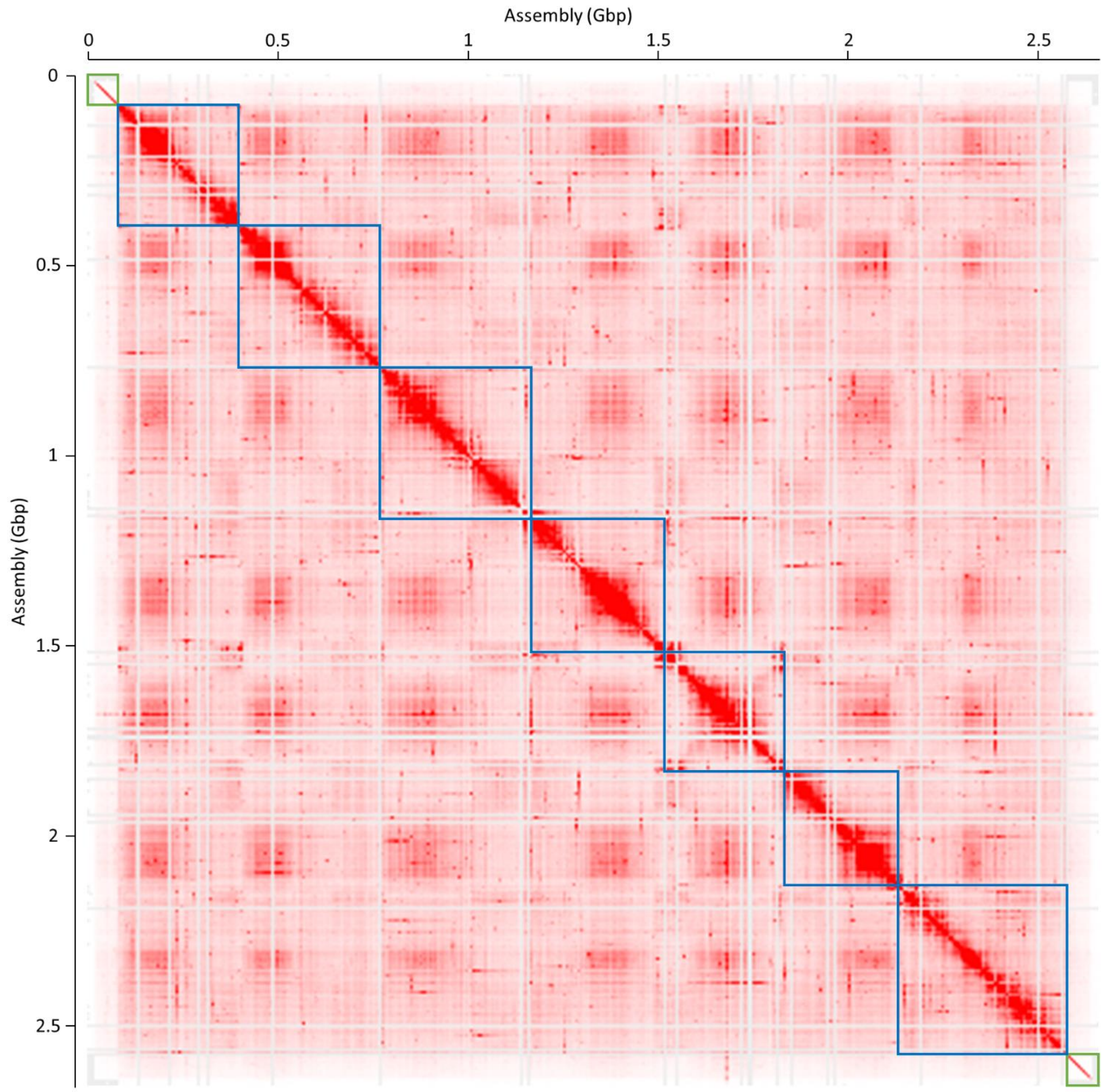

Supplementary Figure 4. HiC contact map showing chromosome-scale scaffolds (blue boxes) and sub-chromosome scale scaffolds (green boxes).

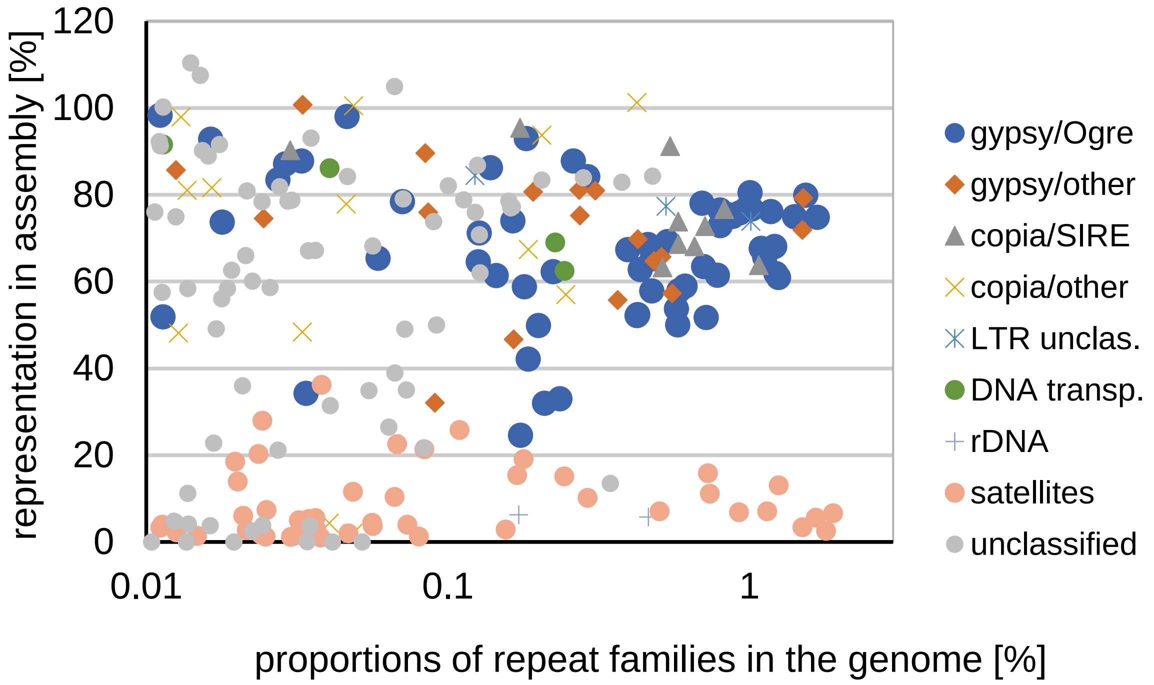

Supplementary Figure 5. Representation of different types of repetitive elements in the assembly. The symbols on the plot represent individual repeat families. The position of each family is determined by its genome proportion determined by the RepeatExplorer2 analysis of unassembled Illumina reads (x-axis), and by its representation in the assembly (y-axis). Repeats with unbiased representation are located at the 100% line, while those below the line are under-represented or absent from the assembly. Source data are provided as a Source Data file.

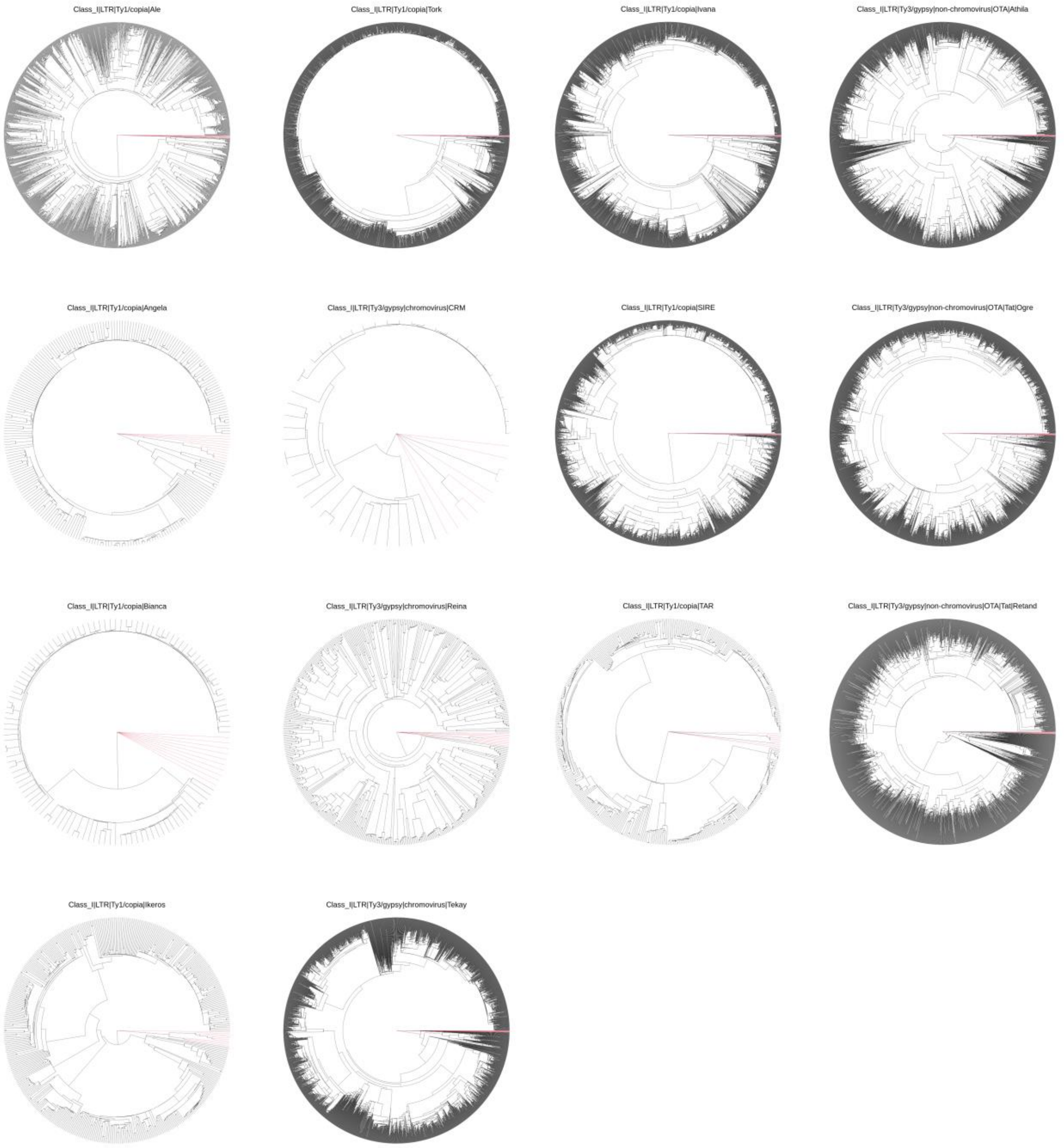

Supplementary Figure 6. Circularised neighbour-joining phylogeny trees of selected Class I TE lineages in the L. sativus genome. These are used to infer age profiles shown in Fig. 2.

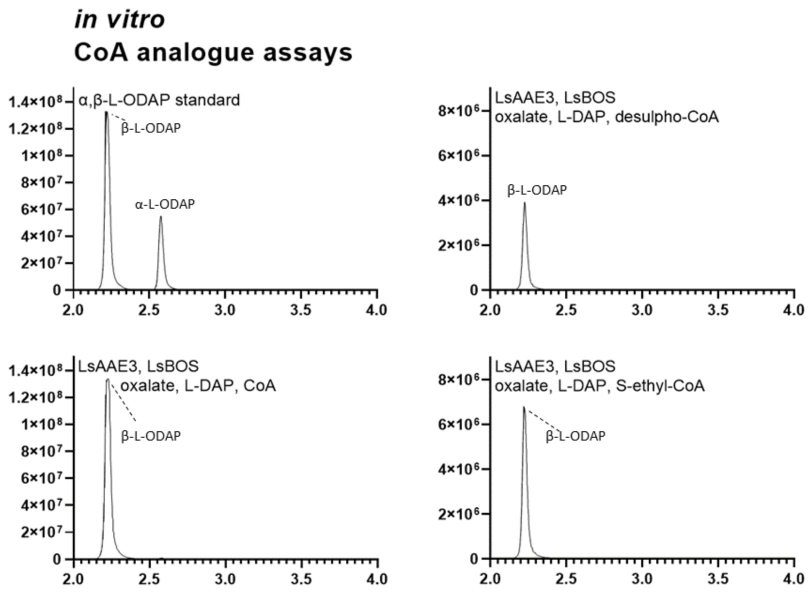

Supplementary Figure 7. LCMS spectra showing β-L-ODAP formation in vitro using using LsAAE3, LsBOS in combination with oxalate, L-DAP and either CoA, desulpho-CoA or S-ethyl-CoA. Source data are provided as a Source Data file.

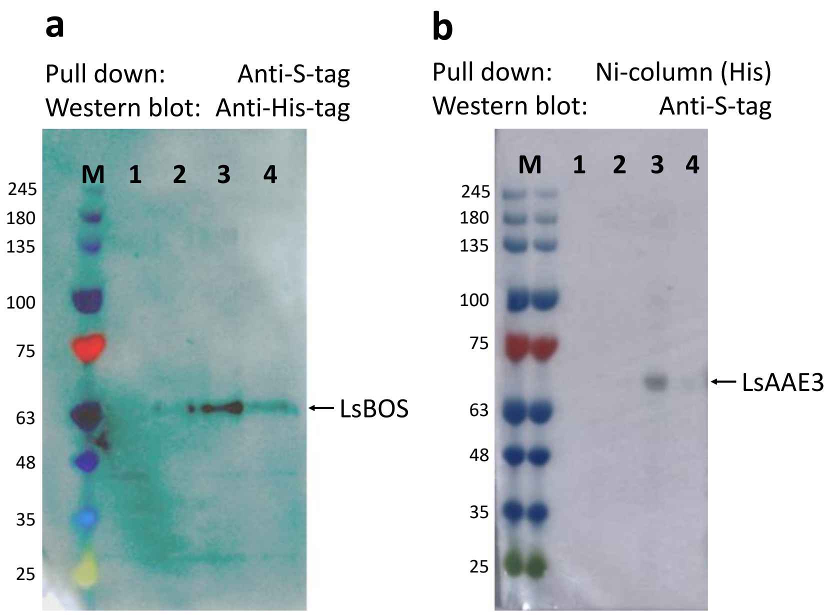

Supplementary Figure 8. Western blots of reciprocal Co-Immunoprecipitation of the LsBOS-LsAAE3 complex following 1h incubation with substrates. The experiment was conducted in triplicate with similar results. a) anti-S-tag antibody pulldown, followed by western blot using an anti-His-tag antibody b) pull-down with a His-tag selective Nickel column, followed by western blot using an anti-S-tag antibody. Both panels: M) Colour Prestained Protein Standard, Broad Range (10-250 kDa), New England Biolabs 1) S-tag LsAAE3, 2) His-tag LsBOS, 3) His-tag LsBOS + S-tag LsAAE3 + L-DAP, 4) His-tag LsBOS + S-Tag LsAAE3 (no L-DAP). Original images provided in a Source Data file.

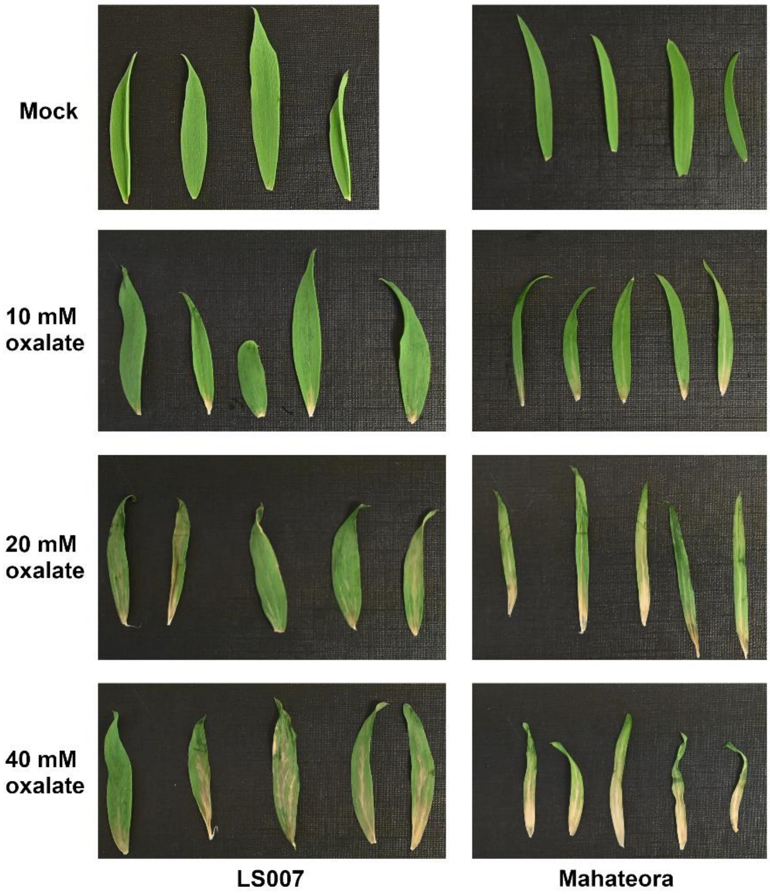

Supplementary Figure 9. Lesions on leaves of grass pea genotypes LS007 (high β-L-ODAP) and Mahateora (low β-L-ODAP) following 24h incubation in oxalate solution or sterile water (all at pH 4.0).

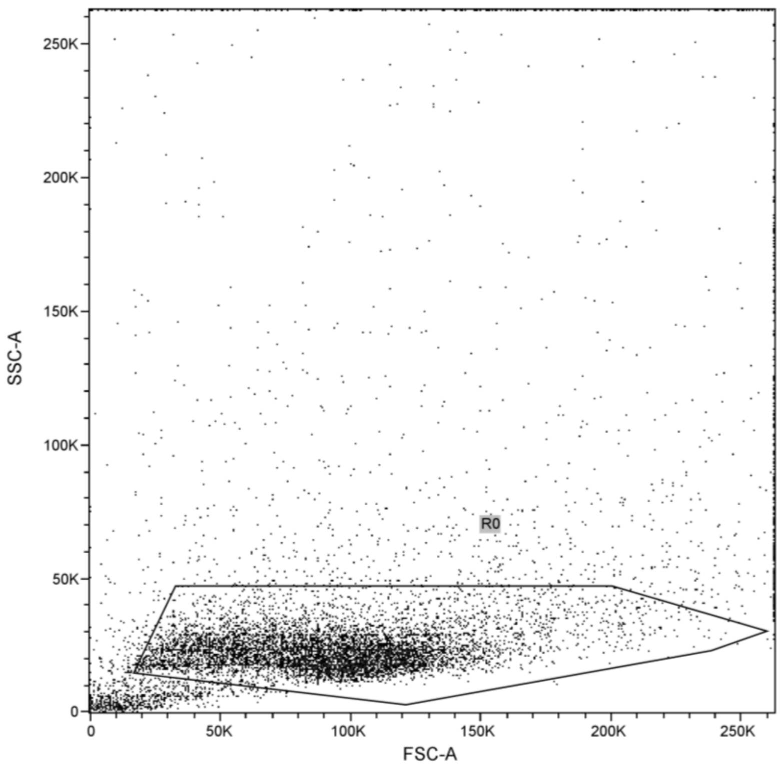

Supplementary Figure 10. Gating strategy used to identify nuclei for genome size estimation shown in Supplementary Figure 1.

a Strong anion exchange chromatography (SAX)

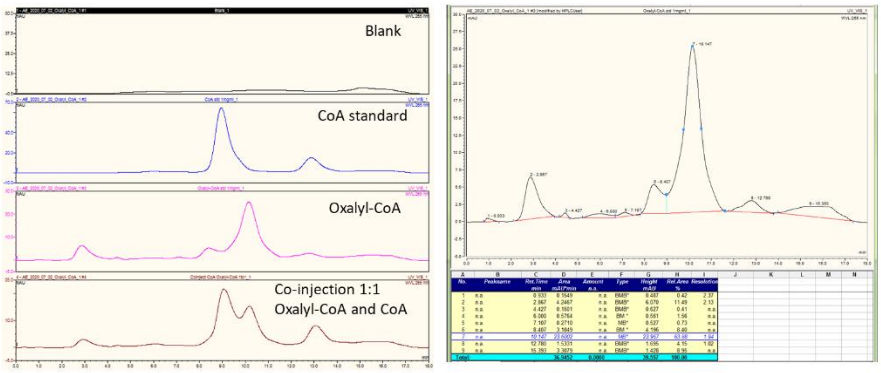

Oxalyl-CoA purity is 64% (peak No 7, Rf 10.1 min)

b MALDI-tof

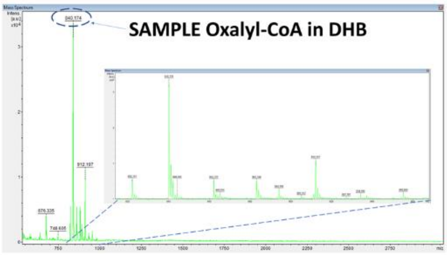

Oxalyl-CoA was detected by MALDI-tof/DHB m/z 840.174 $[M+H]^+$

Supplementary Figure 11. Confirmation of the identity of the oxalyl-CoA used in the *in vitro* enzyme assays. a Strong anion exchange (SAX) chromatography of chemically synthesised oxalyl-CoA. **b** MALDI-ToF mass spectrum of chemically synthesised oxalyl-CoA.

Supplementary Table 1. ONT PromethION sequencing yield.

Flowcell/Nuclease flush	DNA preparation	Library amount (ng)	Read N50 (kbp)	Yield (Gbp) (passed)
FC1 1st load	CN-AMP	250	7.84	51.84
FC1 2nd load	CN-NS-AMP	250	5.25	44.41
FC1 3rd load	QD-SRE	250	18.78	14.28
FC2	QD-SRE	250	22.84	57.48
FC3 1st load	CN-NS-AMP-SRE	270	22.31	41.7
FC3 2nd load	Mix of CN-NS-AMP-SRE & QD-SRE	324	22.81	23.04
FC4	CN-NS-AMP-SRE	400	29.08	32.7
FC5	CN-NS-AMP-SRE	400	24.71	30.7

Supplementary Table 2. LS007 Rbp assembly statistics.

	polished Redbean assembly (Rbp)
A	1,889,610,210
T	1,924,885,321
G	1,230,910,845
C	1,191,840,302
N	0
ATGC bases	6,237,246,678
Total length	6,237,246,678
GC fraction	38.80%
Longest scaffold/contig	2,768,903
N50	157,998
L50	8,679
No. of scaffolds/contigs	162,994

Supplementary Table 3. BlobTools read mapping.

	Strepto-phyta	Proteo-bacteria	Chor-data	Ascomy-cota	Arthro-poda	Firmi-cutes	Eucaryotes (undefined)	no blast hit	not mapped to assembly
Illumina reads (%)	82.6	0.06	0.04	0	0	0	0	8.07	9.22

Supplementary Table 4. AUGUSTUS Gene model summary statistics for the LS007 Redbean assembly.

Gene Build - Augustus	Augustus_Run1	Augustus_Run2	Augustus_Run3
Gene count	98,422	75,695	74,162
Total transcripts	103,879	78,932	79,191
Transcripts per gene	1.06	1.04	1.07
Number of monoexonic genes	37,692	28,018	27,848
Monoexonic transcripts	38,538	28,215	28,069
Transcript mean size cDNA (bp)	1,239.94	1,160.46	1,142.18
Transcript median size cDNA (bp)	975	897	886
cDNA minimum size (bp)	31	28	93
cDNA maximum size (bp)	12,548	12,548	16,258
Total exons	323,386	274,911	295,504
Exons per transcript	3.11	3.48	3.73
Exon mean size (bp)	398.3	333.19	306.09
CDS mean size (bp)	256.62	245.8	241.31
Transcript mean size CDS (bp)	740.63	800.66	849.28
Transcript median size CDS (bp)	534	576	597
CDS minimum size (bp)	5	5	6
CDS maximum size (bp)	12,344	12,344	15,720
Intron mean size (bp)	712.09	612.69	601.6
5' UTR mean size (bp)	215.25	152.65	125.01
3' UTR mean size (bp)	284.06	207.15	167.9

Note: Gene models are informed by transcript assemblies (Supplementary Table 7) and alignments of reference proteins (Supplementary Table 8).

Supplementary Table 5. Total RNA-seq reads for LS007, Mahateora, and LSWT11 and summary of their alignment against the LS007 Rbp genome assembly using HISAT2.1.0.

	LS007	Mahateora	LSWT11
Number of samples	12	12	7
Number of filtered reads	622,496,900	624,553,164	1,299,302,860
Mean fil. rds. per sample	51,874,742	52,046,097	185,614,694
Aligned reads (HISAT2)	536,252,111	525,382,372	1,087,092,030
Aligned reads percentage	86.15%	84.12%	83.67%

Supplementary Table 6. Illumina transcript assembly statistics showing Cufflinks and StringTie assembly results.

Method	Transcripts	Mean number of exons	Mean cDNA size	Number of monoexonic transcripts
StringTie	1575129	4.57	1316.37	362620
Scallop	2693817	3.66	1281.26	981965

Note: Assemblies were run using the 31 alignments produced by HISAT2 (one for each sample), shown in Supplementary Table 5. The transcript number shown represents the total number (redundant set) of transcripts each assembler generated. For each tool, assembled transcripts have been clustered into loci using cuffcompare (cufflinks v2.2.1; command line options "-C -G").

Supplementary Table 7. Mikado transcript assembly statistics for the LS007 Rbp assembly.

Method	Loci	Transcripts	Mean number of exons	Mean cDNA size	Number of monoexonic transcripts
Mikado	42,628	84,837	5.80	1,695.03	5,948

Note: Mikado unifies data generated by the assemblers (Stringtie and Scallop) for each of the 31 alignments one non-redundant set of transcripts (assemblies are summarized in Supplementary Table 6).

Supplementary Table 8. Classification of Mikado transcripts.

Classification	Definition
Gold	Mikado transcripts having a full length hit (complete/putative complete) with full_lengther_next and a maximum of and with at most two complete five_prime_UTRs and three five_prime_UTRs and at most one complete three_prime_UTR and two three_prime_UTRs
Silver	Remaining Mikado transcripts with CDS length >= 900bps and with at most two complete five_prime_UTR's and three five_prime_UTR's and at most one complete three_prime_UTR and two three_prime_UTR'
Bronze	Any remaining Mikado transcripts with defined CDS were assigned to bronze

Supplementary Table 9. Reference protein datasets used with AUGUSTUS.

	Cicer arietinum	*Cucumis sativus*	*Fragaria vesca*	*Glycine max*	*Malus domestica*	*Medicago truncatula*	*Prunus persica*	*Phaseolus vulgaris*	*Trifolium pratense*
Total proteins	33,107	30,364	32,831	88,647	63,517	62,319	47,089	36,995	41,297
proteins aligned	22,890	14,575	7,399	53,077	18,209	32,739	20,221	22,653	24,122
Proteins aligned %	69.1%	48.0%	22.5%	59.9%	28.7%	52.5%	42.9%	61.2%	58.4%
Protein alignments	55,133	37,114	19,767	128,682	51,884	88,027	49,910	56,122	70,681

Note: Proteins were filtered at 50% identity and 80% coverage. Any intron over 10 kbp resulted in the protein alignment being removed.

Supplementary Table 10. Weighting and priorities used in Run3 of gene model generation.

Evidence	Source (for augustus extrinsic config)	Priorities		
		Run1	Run2	Run3
Mikado Transcript Gold	M	10	10	10
Mikado Transcript Silver	F	9	9	9
Mikado Transcript Bronze	E	8	8	8
Mikado Transcripts (all, incl. no CDS)	E	7	7	7
Portcullis pass score = 1 (Gold)	E	6	6	6
Portcullis pass score <1 (Silver)	E	4	4	4
Cross-species protein alignments	P	4	4	9
RNA-Seq coverage hints	W	3	n/a	n/a
Repeats	RM	1	1	1

Supplementary Table 11. Repeat proportions in *Lathyrus sativus* genome (%).

Repeat type							*L. sativus* (Macas *et al.*[1])	*L. sativus* LS007	*L. sativus* LS007
						Analysis	Illumina / RepeatExplorer		assembly annotation
rDNA							1.69	0.59	0.03
satellite							10.73	8.12	1.36
Mobile element	Class I	LTR_unclassified					5.06	2.44	11.17
		Ty1/copia	SIRE				6.85	6.84	8.35
			Ale				0.02	0.11	0.36
			Angela				0.20	0.26	0.00
			Ivana				0.29	0.65	1.35
			Ikeros				0.00	0.00	0.60
			TAR				0.07	0.12	0.00
			Tork				0.33	0.52	1.98
			unclass.				0.00	0.00	0.97
		Ty3/gypsy	Non-chrom.	OTA	Athila		3.11	3.87	3.28
					Tat (other)		0.51	0.18	2.63
						Ogre	45.46	37.32	31.77
			Chromovirus				3.33	4.55	7.64
			unclass.				0.00	0.00	8.20
	Class II	Subclass I	TIR	EnSpm_CACTA			0.03	0.75	0.56
				hAT			0.01	0.03	0.00
				MuDR_Mutator			0.03	0.18	0.33
		Subclass II	Helitron				0.00	0.11	0.00
unclassified/no_evidence							2.67	4.13	0.03
tandem							0.86	0.00	0.00
TOTAL							81.25	70.78	80.61

Supplementary Table 12. Primer sequences.

Species	Gene	Primer name	Sequence (5'->3')
Lathyrus sativus	BOS	BOSF	ATGAGTTCCATCCAAATCCTCTCCAC
		BOSR	TCAACCAGAAGCAGCATCCATAAAC
Lathyrus sativus	AAE3	LsAAE3F	ATGGAAACCGCAACCACCCTCAC
		LsAAE3R	TCAAACTTTAGAAACAAAGTGTTC
Medicago truncatula	BAHD3 (BOS homologue)	MtBAHD3F	ATGAGTTCCATCAAAATCCTCTCCAC
		MtBAHD3R	TTAGTAGGACACAACATCCATAAAC
Medicago truncatula	AAE3	MtAAE3F	ATGGAAACCGCTACAACCCTCAC
		MtAAE3R	TCAAGCTTGAGAGACAAAGTGTTC

Note: Gene-specific primer sequences (not including Gateway cloning sequences) used for Gateway cloning from cDNA.

Supplementary Method 1. Identification of repeats from the genome assembly.

Repeat analysis for the assembly described in this manuscript was carried out using the DANTE pipeline, as described in the Methods section. An additional repeat annotation step was carried out using RepeatModeler as part of the gene annotation pipeline as described below. Both repeat annotations are provided as separate annotation tracks.

RepeatModeler (v1.0.10 - http://www.repeatmasker.org/RepeatModeler/) was used for *de novo* identification of repetitive elements from the assembled grass pea genome sequence. Protein coding genes in the RepeatModeler generated library were hard-masked (i.e. replaced with Ns) using the Arabidopsis Araport11_Release_201606 dataset and *Cicer arietinum* Annotation 101 (https://www.ncbi.nlm.nih.gov/genome/annotation_euk/Cicer_arietinum/101/) coding genes. Any genes with descriptions indicating “transposon” or “helicase” were removed. TransposonPSI (r08222010)[2] was run and significant hits hard-masked, with the output being used to mask the RepeatModeler library. Unclassified repeats were searched in a custom BLAST database of organellar genomes (mitochondrial and chloroplast sequences from Fabales in NCBI nucleotide division, downloaded on 22.9.2017). Any repeat families matching organellar DNA were also hard-masked.

Repeat identification was refined by running RepeatMasker v4.0.7[3] with RepBase Viridiplantae library and with the customized RepeatModeler library (i.e. after masking out protein coding genes), both using the -nolow setting.

The combined masking resulted in 70% of the assembly being soft-masked (i.e. rendered in lowercase to stop alignment of transcriptome data).

Supplementary Method 2. Reference guided transcriptome reconstruction.

Alignment of Illumina RNA-Seq data

RNA-Seq data from three different genotypes LS007, LSWT11 and Mahateora[4,5] was used for grass pea genome annotation: 12 samples from LS007 and Mahateora each (each root and shoot tissues from 3 biological replicates of droughted and non-droughted treatments) and 7 samples from seedling shoot tip, seedling root tip, whole root, whole leaves, early flowers, early pods and late pods from LSWT11 for a total of 31 individual RNA-seq samples). In total, the three filtered datasets comprised over 2.5 billion paired-end reads. For each dataset, read samples were collapsed by tissue and filtered using trim-galore v.0.3.7[6]. Due to concerns about high concentrations of ribosomal RNA, datasets were further filtered using SortMeRNA v. 2.0[7], and using RFam (5S and 5.8S) and Silva (Archaea 16S-23S, Bacteria 16S-23S, Eukaryota 18S-28S) as databases.

Alignment with HISAT2

Filtered reads were aligned to the genome assembly using HISAT2 v2.1.0[8] with option --dta. The RNA-seq data and alignments are summarised in Supplementary Table 5.

Transcript assembly

The Illumina RNA-Seq alignments (31 RNA-Seq transcript alignments from HISAT2) were re-assembled using StringTie2[9] v2.1.1 and Scallop v0.10.2[10], with option --library_type unstranded. The results of transcript assembly are shown in Supplementary Table 6. RNA-Seq junctions were derived from RNA-Seq alignments using Portcullis v1.1.2[11] with default filtering parameters. Junctions that passed the Portcullis filter with a score of 1 were classified as 'Gold' and with a score <1 were classified as 'Silver'.

Mikado v2.0prc2[12], was used to integrate the transcript assemblies generated using StringTie and Scallop (see Supplementary Table 7). Loci were first defined across all assemblies, followed by scoring transcripts based on i) intrinsic metrics relating to ORFs predicted using prodigal v2.6.3 with options -g 1 -f gff[13] (this takes into account ORF and cDNA length, position of the ORF within the transcript, presence of multiple ORFs and UTR lengths), and ii) extrinsic metrics derived from BLASTX searches against the cross-species reference protein database using diamond v0.9.24[14] (with options: blastx --outfmt 6 qseqid sseqid pident length mismatch gapopen qstart qend sstart send evalue bitscore ppos btop) and junctions passing Portcullis filtering. The primary Mikado models for each locus were classified into three categories (Gold, Silver and Bronze) based on their full_lengther next v20131015[15] hits, CDS length and presence of complete UTRs (see Supplementary Table 8).

Supplementary Method 3. Gene prediction using evidence guided AUGUSTUS.

Protein coding genes were predicted using AUGUSTUS[16,17], which uses a Generalized Hidden Markov Model (GHMM) employing both intrinsic and extrinsic information.

Cross-species protein alignment

Predicted protein sequences from 9 species (*Cicer arietinum, Cucumis sativus, Fragaria vesca, Glycine max, Malus domestica, Medicago truncatula, Prunus persica, Phaseolus vulgaris, Trifolium pratense*) were soft-masked for low complexity regions using tantan v22[18] (option: -p) and aligned to the soft-masked grass pea genome sequence (using repeatmodeler repeats) with exonerate v2.4.0[19] with parameters: --model protein2genome --showtargetgff yes --showvulgar yes -M 3936.0 -D 3936.0 --hspfilter 100 --softmaskquery yes --softmasktarget yes --bestn 10 --minintron 20 --maxintron 100000 --showalignment no --geneseed 50 --percent 30 --score 50 –ryo. >%qi\tlength=%ql\talnlen=%qal\tscore=%s\tpercentage=%pi\nTarget>%ti\tlength=%tl\talnlen=%tal\n. Exonerate alignments were filtered to two criteria a high confidence alignment set, (50% identity and 100% coverage) and a more comprehensive set (50% identity and 80% coverage) for use with AUGUSTUS. Mikado was used to create gene models from the protein alignments, with UTRs added based on the transcriptome assemblies (StringTie and Scallop). Alignments with introns longer than 10 kbp were removed from further analyses (see Supplementary Table 9).

Gene predictor training

The Mikado gold set transcripts were selected for training AUGUSTUS[20]. We excluded genes with a genomic overlap within 1000bp of a second gene and gene models that were homologous to each other with coverage and identity ≥ 80%. The filtered set contained 9604 transcripts, from which 2000 transcripts were selected at random for training AUGUSTUS and another 200 transcripts were used for testing. The trained AUGUSTUS model resulted in values of 0.976 sensitivity (sn), 0.909 specificity (sp) at the nucleotide level, sn 0.837, sp 0.801 at the exon level and sn 0.41, sp 0.376 at the gene level.

Gene model generation

AUGUSTUS 3.3.3 was used to predict gene models for the assembly using the evidence hints generated from nine sets of cross species protein alignments (listed above), Mikado Illumina models and intron/exon junctions defined using the RNA-Seq data. AUGUSTUS options were set to: --AUGUSTUS_CONFIG_PATH=config --species=Lathyrus_sativus --UTR=on --extrinsicCfgFile=extrinsic.ei_augustus333_generic.cfg --alternatives-from-evidence=true --hintsfile=extrinsic.augustus.run{1,2,3}.gff --noInFrameStop=true --allow_hinted_splicesites=atac. Interspersed repeats were provided as “nonexonpart” to exclude them from analysis. AUGUSTUS was run three times, with different assigned additional bonus scores and priority based on evidence type and classification (Gold, Silver, Bronze) to reflect the reliability of different evidence sets:

- Run1 utilized the evidence hints generated from Mikado transcript models, RNA-Seq Portcullis junctions, cross-species protein alignments (filtered at 80% coverage and 50% identity), RNA-Seq read coverage and interspersed repeats by using the sources and priorities described in Supplementary Table 10.
- Run2 used same evidence hints as Run1, except for RNA-Seq read coverage hints, which were not included.

- Run3 used same evidence hints as Run2, but with higher weightage to the cross-species protein alignments and changed priorities as described in Supplementary Table 10.

Gene model evaluation

The final set of gene models was selected using Minos-Mikado (https://github.com/EI-CoreBioinformatics/minos). The 3 alternative AUGUSTUS models and Mikado gene models derived from both the transcript assemblies and protein alignments were used in Minos and a final set of models selected based on evidence support and intrinsic features of the models. All models were assigned a confidence classification (high, Low) with high confidence defined by a BUSCO assignment of complete or an average homology coverage of at least 80% or at least 60% and also 40% support from transcript data. Models not meeting this definition were assigned as protein coding low confidence unless they fell below an average homology coverage of less than 30% and a coding potential (CPC) score of less than 0.25 in which case they were classified as predicted genes. Any models with greater than 40% overlap with repeat regions were flagged as repeat associated. Models with no evidence support were discarded.

Functional annotation of protein coding transcripts

Proteins were annotated using AHRD v.3.3.3[21]. Predicted protein sequences were BLASTp (v2.6.0, e-value 1e5) searched against *Arabidopsis thaliana* TAIR10protein sequences[22] and the viridiplantae sequences of UniProt v. 11May2020, both SwissProt and TREMBL datasets[23] using BLASTP+ v. 2.6.0 asking for a maximum e-value of 1e-5. We also ran InterProScan 5.22.61[24] and provided the InterProScan output to AHRD. The standard example configuration file pathtest/resources/ahrd_example_input.yml, distributed with the AHRD tool was adapted by i) providing the GOA mapping from UniProt, ii) including the InterPro database, iii) setting parameter 'prefer_reference_with_go_annos' to 'false' and not using the parameter 'gene_ontology_result' and iv) amending the regular expression used for protein fasta headers.A separate blast of the annotated proteins (blastp; -max_target_seqs 1, -evalue 1e-5) was run versus the reference proteins to identify the best blast hit and is provided as part of the functional annotation.

Supplementary references

1. Macas, J. *et al.* In Depth Characterization of Repetitive DNA in 23 Plant Genomes Reveals Sources of Genome Size Variation in the Legume Tribe Fabeae. *PLoS ONE* **10**, e0143424 (2015).
2. Haas, B. TransposonPSI: An Application of PSI-Blast to Mine (Retro-)Transposon ORF Homologies. http://transposonpsi.sourceforge.net/ (2010).
3. Smit, A. F. A., Hubley, R. & Green, P. *RepeatMasker*. (2013).
4. Emmrich, P. M. F. Genetic improvement of grass pea (*Lathyrus sativus*) for low β-L-ODAP content. (University of East Anglia, 2017).
5. Emmrich, P. M. F. *et al.* Linking a rapid throughput plate-assay with high-sensitivity stable-isotope label LCMS quantification permits the identification and characterisation of low β-L-ODAP grass pea lines. *BMC Plant Biol* **19**, 489 (2019).
6. Babraham Bioinformatics - Trim Galore! https://www.bioinformatics.babraham.ac.uk/projects/trim_galore/.
7. Kopylova, E., Noé, L. & Touzet, H. SortMeRNA: fast and accurate filtering of ribosomal RNAs in metatranscriptomic data. *Bioinformatics* **28**, 3211–3217 (2012).
8. Kim, D., Langmead, B. & Salzberg, S. L. HISAT: a fast spliced aligner with low memory requirements. *Nat Methods* **12**, 357–360 (2015).
9. Pertea, M. *et al.* StringTie enables improved reconstruction of a transcriptome from RNA-seq reads. *Nat. Biotechnol.* **33**, 290–295 (2015).
10. Shao, M. & Kingsford, C. Accurate assembly of transcripts through phase-preserving graph decomposition. *Nature Biotechnology* **35**, 1167–1169 (2017).
11. Mapleson, D., Venturini, L., Kaithakottil, G. & Swarbreck, D. Efficient and accurate detection of splice junctions from RNA-seq with Portcullis. *Gigascience* **7**, (2018).
12. Venturini, L., Caim, S., Kaithakottil, G. G., Mapleson, D. L. & Swarbreck, D. Leveraging multiple transcriptome assembly methods for improved gene structure annotation. *Gigascience* **7**, (2018).

13. Hyatt, D. *et al.* Prodigal: prokaryotic gene recognition and translation initiation site identification. *BMC Bioinformatics* **11**, 119 (2010).

14. Buchfink, B., Xie, C. & Huson, D. H. Fast and sensitive protein alignment using DIAMOND. *Nat Methods* **12**, 59–60 (2015).

15. Seoane, P., Fernandez, N. & Guerrero, D. full_lengther_next. (2018).

16. Stanke, M., Steinkamp, R., Waack, S. & Morgenstern, B. AUGUSTUS: a web server for gene finding in eukaryotes. *Nucleic Acids Research* **32**, W309–W312 (2004).

17. Stanke, M. *et al.* AUGUSTUS: ab initio prediction of alternative transcripts. *Nucleic Acids Res* **34**, W435–W439 (2006).

18. Frith, M. C. A new repeat-masking method enables specific detection of homologous sequences. *Nucleic Acids Res* **39**, e23–e23 (2011).

19. Slater, G. S. C. & Birney, E. Automated generation of heuristics for biological sequence comparison. *BMC Bioinformatics* **6**, 31 (2005).

20. Grabherr, M. G. *et al.* Full-length transcriptome assembly from RNA-Seq data without a reference genome. *Nature Biotechnology* **29**, 644–652 (2011).

21. Hallab, A. Protein Function Prediction using Phylogenomics, Domain Architecture Analysis, Data Integration, and Lexical Scoring. (Rheinische Friedrich-Wilhelms-Universität, 2014).

22. Cheng, C.-Y. *et al.* Araport11: a complete reannotation of the *Arabidopsis thaliana* reference genome. *The Plant Journal* **89**, 789–804 (2017).

23. The UniProt Consortium. UniProt: a worldwide hub of protein knowledge. *Nucleic Acids Res* **47**, D506–D515 (2019).

24. Jones, P. *et al.* InterProScan 5: genome-scale protein function classification. *Bioinformatics* **30**, 1236–1240 (2014).
