# Supplementary material for: Genomics and biochemical analyses reveal a metabolon key to β-L-ODAP biosynthesis in Lathyrus sativus
Source: Nat Commun. 2023 Feb 16;14:876. doi: 10.1038/s41467-023-36503-2 (PMC9935904; doi:10.1038/s41467-023-36503-2)
Supplement: Supplementary file 3 — Description of Additional Supplementary Files [file 41467_2023_36503_MOESM3_ESM.pdf]

Description of Additional Supplementary Files

File Name: Supplementary Data 1
Description: Complete phylogeny of BAHD-acyltransferases derived from the LS007 genome assembly, TAIR11 Arabidopsis thaliana and the INRA Pisum sativum v1a genome assembly

File Name: Supplementary Data 2
Description: Annotations corresponding to gene candidates for enzymes involved in β-L-ODAP synthesis
