# Supplementary material for: Genomics and biochemical analyses reveal a metabolon key to β-L-ODAP biosynthesis in Lathyrus sativus
Source: Nat Commun. 2023 Feb 16;14:876. doi: 10.1038/s41467-023-36503-2 (PMC9935904; doi:10.1038/s41467-023-36503-2)
Supplement: Supplementary file 6 — Reporting Summary [file 41467_2023_36503_MOESM6_ESM.pdf]

nature portfolio

Corresponding author(s): Peter MF Emmrich

Last updated by author(s): 31/01/2023

Reporting Summary

Nature Portfolio wishes to improve the reproducibility of the work that we publish. This form provides structure for consistency and transparency in reporting. For further information on Nature Portfolio policies, see our Editorial Policies and the Editorial Policy Checklist.

Statistics

For all statistical analyses, confirm that the following items are present in the figure legend, table legend, main text, or Methods section.

n/a	Confirmed	
☐	☒	The exact sample size (*n*) for each experimental group/condition, given as a discrete number and unit of measurement
☐	☒	A statement on whether measurements were taken from distinct samples or whether the same sample was measured repeatedly
☒	☐	The statistical test(s) used AND whether they are one- or two-sided<br>*Only common tests should be described solely by name; describe more complex techniques in the Methods section.*
☒	☐	A description of all covariates tested
☒	☐	A description of any assumptions or corrections, such as tests of normality and adjustment for multiple comparisons
☐	☒	A full description of the statistical parameters including central tendency (e.g. means) or other basic estimates (e.g. regression coefficient) AND variation (e.g. standard deviation) or associated estimates of uncertainty (e.g. confidence intervals)
☒	☐	For null hypothesis testing, the test statistic (e.g. *F*, *t*, *r*) with confidence intervals, effect sizes, degrees of freedom and *P* value noted<br>*Give P values as exact values whenever suitable.*
☒	☐	For Bayesian analysis, information on the choice of priors and Markov chain Monte Carlo settings
☒	☐	For hierarchical and complex designs, identification of the appropriate level for tests and full reporting of outcomes
☒	☐	Estimates of effect sizes (e.g. Cohen's *d*, Pearson's *r*), indicating how they were calculated

Our web collection on statistics for biologists contains articles on many of the points above.

Software and code

Policy information about availability of computer code

Data collection	Genome size estimation: FACSCantoll flow cytometer firmware; Sequencing: Illumina HiSeq 2500 firmware, ONT PromethION Beta R9.4.1, basecalling: guppy dna_r9.4.1_450bps_hac.cfg; LCMS: Acquity UPLC console build 05 July 2011,Waters MassLynx V4.1; surface plasmon resonance: Biacore 8K (Cytiva) firmware with series S sensor chip CM5
Data analysis	Genome size estimation: FCSalyser (v. 0.9.18 alpha); expression analysis: Trinity v2.1.1, RSEM v1.2.20; genome assembly: redbean v2.2, bwa 0.7.17; genome annotation: RepeatModeler v1.0.10, TransposonPSI r08222010, RepeatMasker v4.0.7, SortMeRNA v. 2.0, HISAT v2.1.0, StringTie2 v2.1.1, Scallop v0.10.2, Portcullis v1.1.2, Mikado v2.0prc2, diamond v0.9.24, Augustus 3.3.3, BLASTp v2.6.0; completeness: BUSCO v.4.0.4_cv1, assembly stats 17.02, BlobTools 1.1.1, Juicer 1.6, 3D-DNA (release 201008-cb63403); BAHD-At phylogeny: InterProScan 5.44-79.0, MUSCLE 3.8, Jalview 2.1, RAxML-8.2.12, iTOL v6; repeat analysis: RepeatExplorer2, DANTE 0.1.1; surface plasmon resonance: Biacore Insight Evaluation Software; statistics and visualisation: R v 3.6.0, RStudio 1.2.1335, Graphpad 9.2.0; Image compositing: GIMP 2.10.8; chemical drawing: MarvinSketch 19.13

For manuscripts utilizing custom algorithms or software that are central to the research but not yet described in published literature, software must be made available to editors and reviewers. We strongly encourage code deposition in a community repository (e.g. GitHub). See the Nature Portfolio guidelines for submitting code & software for further information.

Data

Policy information about availability of data

All manuscripts must include a data availability statement. This statement should provide the following information, where applicable:

- Accession codes, unique identifiers, or web links for publicly available datasets
- A description of any restrictions on data availability
- For clinical datasets or third party data, please ensure that the statement adheres to our policy

BUSCO analyses were done using odb10 databases
All plant materials used in this article are available from the corresponding author. The PromethION and genomic Illumina sequencing datasets used and analysed during the current study have been uploaded to the European Nucleotide Archive under accession PRJEB33571. RNAseq and HiC sequencing datasets are being uploaded to the NCBI Gene Expression Omnibus under accession GSE223956. Genome assembly and annotations are available under DOI 10.5281/zenodo.7390878.

Human research participants

Policy information about studies involving human research participants and Sex and Gender in Research.

Reporting on sex and gender	no human research participants were involved in this study.
Population characteristics	no human research participants were involved in this study.
Recruitment	no human research participants were involved in this study.
Ethics oversight	no human research participants were involved in this study.

Note that full information on the approval of the study protocol must also be provided in the manuscript.

Field-specific reporting

Please select the one below that is the best fit for your research. If you are not sure, read the appropriate sections before making your selection.

☒ Life sciences ☐ Behavioural & social sciences ☐ Ecological, evolutionary & environmental sciences

For a reference copy of the document with all sections, see nature.com/documents/nr-reporting-summary-flat.pdf

Life sciences study design

All studies must disclose on these points even when the disclosure is negative.

Sample size	Sample sizes are described for each experiment. At a minimum, three biological replicates were used. Since the experiments described used seed derived from single-seed descent with minimal genetic variation or clonal strains of bacteria, the described sample sizes were considered sufficient.
Data exclusions	no data was excluded from the analyses
Replication	All biological samples were derived from isogenic populations of seeds, which are available from the John Innes Centre Germplasm Research Unit. At a minimum, three biological replicates were used. All replications were successful.
Randomization	wherever populations were sampled into groups for different treatments, this allocation was random. Plants' positions in growth chambers were also randomised.
Blinding	No human research participants were involved and blinding of researchers was not considered practical as all experiments were performed in a molecular biology/biochemistry laboratory setting, with measurements taken by automatic equipment.

Reporting for specific materials, systems and methods

We require information from authors about some types of materials, experimental systems and methods used in many studies. Here, indicate whether each material, system or method listed is relevant to your study. If you are not sure if a list item applies to your research, read the appropriate section before selecting a response.

Materials & experimental systems

n/a	Involved in the study
☐	☒ Antibodies
☒	☐ Eukaryotic cell lines
☒	☐ Palaeontology and archaeology
☒	☐ Animals and other organisms
☒	☐ Clinical data
☒	☐ Dual use research of concern

Methods

n/a	Involved in the study
☒	☐ ChIP-seq
☐	☒ Flow cytometry
☒	☐ MRI-based neuroimaging

Antibodies

Antibodies used

Primary: 6x-His Tag Mouse Monoclonal Antibody, Thermo Fisher Scientific MA1-21315, lot XD343962 + Secondary: Goat Anti-Mouse IgG (FC Specific), Sigma A0168, Batch 00000962/6; Anti S-Tag Rabbit Monoclonal Antibody, Abcam, ab180958, EPR 12996, lot GR148713-5 + Secondary: Goat Anti-Rabbit IgG (FC Specific) Sigma A0545, Lot 069M4835V.

Validation

Both antibodies used are recombinant antibodies produced in animal-free systems with high batch-to-batch consistencies. Their targets are species-independent peptide tags. Validation and datasheets are available on https://www.thermofisher.com/antibody/product/6x-His-Tag-Antibody-clone-HIS-H8-Monoclonal/MA1-21315, hhttps://www.sigmaaldrich.com/GB/en/product/sigma/a0168, https://www.abcam.com/s-tag-antibody-epr12996-ab180958.html and https://www.sigmaaldrich.com/GB/en/product/sigma/a0545 respectively.

Flow Cytometry

Plots

Confirm that:

☒ The axis labels state the marker and fluorochrome used (e.g. CD4-FITC).

☒ The axis scales are clearly visible. Include numbers along axes only for bottom left plot of group (a 'group' is an analysis of identical markers).

☒ All plots are contour plots with outliers or pseudocolor plots.

☒ A numerical value for number of cells or percentage (with statistics) is provided.

Methodology

Sample preparation

Cell nuclei were isolated from leaf samples of grass pea and pea, as described by Dolezel et al. (2007). Nuclei from pea and grass pea were mixed together following staining with propidium iodide.

Instrument

FACSCantoll Becton Dickinson

Software

FCSalyser v. 0.9.18 alpha

Cell population abundance

No sorting was undertaken. Events were SSC/FCS gated (see figure in supplementary information) and assigned as pea or grass pea according to peaks in the event count distribution of PE.A intensity. In each of three replicates a mean of 2396 events were assigned as pea and 1523 as grass pea.

Gating strategy

Events of SSC-A between 10k and 50k, and FSC-A > 15k were included in the analysis. Events in the PE.A peak at 98k were assigned as pea, events in the peak at 145k were assigned as grass pea.

☒ Tick this box to confirm that a figure exemplifying the gating strategy is provided in the Supplementary Information.
